# “Somewhat of an Adult”: Understanding the “Dance” of Competing Tensions Parents Manage While Caring for an Adolescent or Young Adult (AYA) Diagnosed with Hematologic Malignancy

**DOI:** 10.3390/cancers17081299

**Published:** 2025-04-12

**Authors:** M. Devyn Mullis, Carma L. Bylund, Diliara Bagautdinova, Emma G. Bryan, Maria Sae-Hau, Elisa S. Weiss, Joanne P. Lagmay, Carla L. Fisher

**Affiliations:** 1 Department of Health Outcomes & Biomedical Informatics, University of Florida, Gainesville, FL 32610, USA; mullis.md@ufl.edu (M.D.M.); carma.bylund@ufl.edu (C.L.B.); emma.bryan@ufl.edu (E.G.B.); 2 Department of Oncology, Karmanos Cancer Institute, Wayne State University School of Medicine, Detroit, MI 48201, USA; hs1330@wayne.edu; 3 The Leukemia & Lymphoma Society, Washington, DC 20005, USA; maria.sae-hau@lls.org (M.S.-H.); elisa.weiss@lls.org (E.S.W.); 4 Department of Pediatrics, Pediatric Hematology/Oncology, University of Florida, Gainesville, FL 32610, USA; jplagmay@ufl.edu

**Keywords:** adolescents and young adults, parent caregivers, caregiving, blood cancer, dialectical tensions, qualitative research, family communication, healthcare communication

## Abstract

Background: Parents supporting AYAs with blood cancer juggle dual, competing roles as cancer caregiver and parent, which may heighten distress as they feel pulled simultaneously in two opposing directions. Likewise, AYAs encounter paradoxical needs as they revert to being more dependent on their parents to prioritize their survival while their developmental trajectory toward independence is disrupted. Parents need help understanding the underlying tensions they face in caregiving to reduce their distress and promote their connectedness with their AYA. Using a dialectical lens, we identified tensions parents encountered while caregiving in three contexts (clinical, family, and online communication) to inform a targeted psychosocial intervention. Methods: In partnership with The Leukemia & Lymphoma Society, we recruited 20 parents for in-depth interviews. Parents cared for adolescents aged 15–18 (*n* = 10) or emerging adults aged 19–29 (*n* = 10) diagnosed >3 months prior and in active treatment or within 2 years since treatment ended. Transcripts were thematically analyzed. Results: Parents described four ongoing tensions they needed to negotiate as they cared for their AYA: (1) *being the driver versus passenger in their child’s care*; (2) *coping with cancer together as a family versus separately*; (3) *deciding to reveal versus conceal information*; and (4) *expecting normative developmental and disease trajectories versus disrupted trajectories*. These tensions characterize the complex caregiving “dance” parents navigate in all three care contexts. Conclusions: Psychosocial education can normalize these tensions for parents to promote healthier coping and reduce distress while enhancing connectedness with their AYA. As caregiver–patient outcomes are interrelated, it may improve AYAs’ well-being.

## Simple Summary

Parents are often the primary caregiver and source of support for their adolescent or young adult living with blood cancer. In these roles they navigate tensions or competing needs in online, clinical, and family care contexts. This experience can feel like being pulled in two directions. Parents described four tensions that illustrate being pulled in two directions simultaneously, tensions they must constantly navigate as they care for and cope with their child: (1) being the driver versus passenger in their child’s care; (2) coping with cancer as a family versus individually; (3) deciding whether to reveal versus conceal information; and (4) expecting typical developmental or disease experiences for their child versus managing unexpected experiences. These findings can be used as the bedrock of resources for parents to help normalize the conflicting realities with which parents cope in their cancer caregiving role and foster health-promoting approaches to navigating these tensions.

## 1. Introduction

Parents are oftentimes the primary caregiver and source of support for their adolescent or young adult (AYA) living with hematologic malignancy (blood cancer)—the most common cancer type diagnosed from childhood through young adulthood [1]. Caregiving parents of AYAs report significant levels of distress, including symptoms of post-traumatic stress disorder, and their diagnosed AYAs report similar outcomes indicating their distress is interrelated [2,3,4]. Parents’ distress is not surprising given the threat to their child’s life in addition to blood cancer caregiving being especially turbulent. In comparison to solid tumor cancers, blood cancer care demands can be characterized by immediate and long-term hospitalizations, years of or indefinite costly treatment(s), multiple treatment transitions, travel or relocation for treatment, employment and/or financial hardship, stress on the family’s functioning, as well as challenging physical, psychological, and behavioral treatment side effects [5,6,7]. These care stressors are compounded by the trauma of an AYA facing a life-threatening disease so early in the lifespan, when they are just beginning to develop a sense of self, independence, and separation from their parent [8,9]. Like their caregiving parents, AYAs report significant psychological distress and are at a higher risk of distress, depression, and anxiety in comparison to older adults living with cancer [10]. 

Diagnosed AYAs often look to their parents as their primary source of support in navigating this traumatic life experience within a complex healthcare system. While supportive care resources for family caregivers in acute or chronic illness are scarce [11], caregiving support for parents is especially limited in AYA oncology care [12]. A recent review showed that family caregivers need supportive resources to help them manage distress by enhancing their caregiving ability and that caregivers especially want education on what to expect in their role and how to navigate it relationally with their diagnosed loved one [11,12]. 

Parents of diagnosed AYAs would benefit from a “psychosocial map” of psychosocial support and education that teaches them about the “oppositional tensions”—feeling pulled in two directions simultaneously—that they must learn to navigate while parenting and caring for an AYA with blood cancer [13,14]. A dialectical perspective in both psychotherapy practice and behavioral research highlights the importance of normalizing for individuals and families that dual or competing forces define their illness experiences and that it is important to make sense of the “parts” to navigate the “whole” [13]. This cognitive processing or sense-making should be embedded within the relational communication and care contexts in which the tensions emerge, such as online, clinical, or family communication contexts of care [13]. Studies demonstrate that when individuals can identify, acknowledge, and reconcile these tensions, they can embrace a more adaptive mindset and flexible coping approach that is tied to better emotion regulation and less distress [15,16].

Both caregiving parents and their AYAs characterize their cancer experience as fraught with paradoxes, though this research has typically centered on tensions encountered in individuals’ coping experiences and not within caregiving contexts. For instance, parents describe themselves simultaneously living in fear of their child’s survival while trying to be positive and hopeful, especially in their child’s presence [17,18,19]. Likewise, AYAs describe themselves longing for their pre-cancer self while grappling with their identity as patient or survivor taking center stage [20]. Research informed by a relational dialectics theoretical approach (RDT) has helped unveil and normalize the conflicting nature of illness caregiving and coping at the relational level as caregivers and patients co-navigate multiple and, at times, competing roles (e.g., cancer caregiver versus parent of an AYA; patient versus son/daughter) as they take on the illness together. These studies have highlighted the contradictory nature of living with various types of cancers by identifying common discursive struggles—referred to as dialectical tensions—that parents and family members often encounter, like dualities of autonomy–control, openness–closedness, connection–separation, and the individual–collective [21,22,23,24,25,26]. 

In using a dialectical approach, we can help normalize the distinct paradoxical experience of AYA caregiving for both parents and AYAs—evidence that can become the bedrock of future interventions. Psychosocial interventions developed or adapted for specific types of caregivers are needed to reduce their psychological distress by providing them with a roadmap of targeted guidance for their unique circumstances, like the disease type (e.g., blood cancers) and relationship type (e.g., parent–child), as well as the phase of the lifespan in which cancer is diagnosed (e.g., AYA) [27,28]. A recent cancer caregiver intervention (*Healthy Communication Practice*) addresses and normalizes common tensions that emerge in the parent–child bond (e.g., independence–dependence) to help adult child caregivers develop communication skills central to their caregiving role. This is done in three communication contexts in which they engage in caregiving: online, clinical, and family care contexts [29,30,31]. The online intervention was both feasible and acceptable for adult child caregivers of diagnosed aging parents living with a blood cancer [29], while also effectively reducing caregivers’ distress and improving their communication skills [31]. To adapt and target this intervention for parents of AYAs, we aimed to capture the tensions parents encounter in their role as a caregiver to their AYA living with a blood cancer in each care context to help parents identify, acknowledge, and reconcile these tensions that can complicate their role in the online, clinical, and family communication contexts of caregiving.

## 2. Materials and Methods

### 2.1. Sample 

In partnership with The Leukemia & Lymphoma Society (LLS), parents caring for an AYA were recruited via email through LLS’s constituent database to participate in an individual, in-depth interview. Inclusion criteria were (1) a parent caring for adolescent aged 15–18 or emerging adult aged 19–29 diagnosed >3 months prior with a blood cancer in active treatment or within 2 years since treatment ended, and (2) English-speaking. Interested parents could contact a research coordinator via a link in the email to screen for eligibility and schedule an interview.

### 2.2. Procedures

In-depth, semi-structured interviews with parent caregivers were conducted and audio-recorded (July–September 2024). The interview guide was previously created and tested in formative research to develop the *Healthy Communication Practice* intervention and then adapted and expanded upon to target the parent caregiver of a diagnosed AYA for intervention adaptation to provide targeted guidance for communication skill development in three communication contexts of care (online, clinical, and family communication) (see [32]). C.L.F. and M.D.M. led the development of the guide, which involved experts in family communication (C.L.F.; M.D.M.; D.B.; C.L.B.), clinical communication (C.L.F.; C.L.B.; D.B.), online communication (C.L.B.), cancer caregiving (C.L.B.; C.L.F.), and blood cancer (E.W.; M.S.; J.P.L.). Participants were asked about various caregiving experiences (e.g., diagnosis, clinical communication, family support). In each communication context, parents were asked about their role, challenges faced, as well as coping responses (e.g., *How do you see your role at your child’s clinical appointments? What challenges have you encountered in your caregiving role at your child’s clinical appointments? How have you managed this?)*. No direct questions were asked about dialectical tensions (e.g., control–autonomy), which emerged naturally in parents’ characterizations of their experiences. On average, interviews lasted 92 min (ranging 25–200 min). Transcriptions resulted in 472 single-spaced pages of data. Participants were compensated with a $50 gift card.

### 2.3. Data Analysis

A thematic analysis was conducted using a constant comparative method (CCM) approach [33] with RDT as a sensitizing framework to capture the dialectical tensions parents encounter in three caregiving contexts (online, clinical, and family communication). To identify tensions, the analysis was segmented or kept separate for each communication care context to compare emergent tensions in each context. ATLAS.ti 24.1.1 was used to manage the data. A multi-coder approach and triangulation of stakeholder perspectives (i.e., parents caring for EA versus adolescent) were used to promote rigor in analysis. The lead author and qualitative analyst with expertise in RDT (MDM) led the analysis by inductively analyzing all transcripts. The senior author and lead qualitative expert on the team, also with expertise in RDT (CLF), served as a second coder to validate analysis and co-develop a codebook over the course of multiple consensus meetings. The CCM steps were followed in analysis: (1) open coding to find patterns using contextual labels; (2) collapsing emergent patterns to develop themes; and (3) axial coding to further define themes and identify thematic properties using RDT typologies to further define tensions (e.g., control–autonomy). The extent of thematic saturation was identified using Owen’s criteria of repetition, recurrence, and forcefulness [34]. Moreover, the sample size (*n* = 20) exceeds sample sizes used in studies of the same design where saturation is reached, demonstrating extended rigor in this project [35]. To capture similarities and differences in parents’ experiences, transcripts for parents of adolescents were kept separate from emerging adults, and typologies were later collapsed given significant overlap in parents’ experiences.

## 3. Results

Twenty parents participated and were mostly mothers (95%) caring for either a son (75%) or daughter (25%), with half in emerging adulthood (19–29) and half in adolescence (15–18). The majority of their AYAs were diagnosed with a leukemia (75%). Most parents were non-Hispanic white (80%), with 50% working full-time. See Table 1 for full demographics.

Parents described four ongoing dialectical tensions—feeling pulled in two directions simultaneously—as they cared for their AYA: (1) *being the driver versus passenger in their child’s care*; (2) *coping with cancer together as a family versus separately*; (3) *weighing whether to reveal versus conceal information;* and (4) *expecting normative developmental and disease trajectories versus disrupted trajectories*. These tensions illustrate the complex caregiving “dance” parents must learn to manage in all three care contexts, particularly clinical and family communication contexts of care. Each tension is labeled within the AYA oncology care context and then defined using common RDT constructs that identify the opposing yet unified forces (e.g., control–autonomy). These are also underlined to further illustrate the tension parents face in their AYA oncology care role. Each tension is then depicted in each caregiving context (e.g., online, clinical or family communication) in which parents described the tension using their lived narratives to more comprehensively capture the dialectical pulls parents negotiate across different contexts of care. A translational table (Table 2) also presents the typology of tensions using action statements, a technique that facilitates the translation of findings into practice and interventions to provide supportive care to parents [36,37].

### 3.1. Being the Driver Versus Passenger in Their Child’s Cancer Care 

Parents described a paradox of being the leader (i.e., driver) of their AYA’s care as their caregiver, while also functioning as a passenger, albeit sometimes reluctantly. At times, parents were (or wanted to be) in control of their child’s care. Yet, concurrently parents wanted to promote (or needed to respect) their AYA’s autonomy in their decision making. Being a driver versus a passenger in their child’s care was especially complex given their child’s developmental phase. As one parent described it, “It’s a dance. … He’s somewhat of an adult” (1).

#### 3.1.1. Online

A parent explained how this tension characterized her experience with online information management and sharing. While she wanted to control her AYA’s access to online information, at the same time, she acknowledged being a passenger in that experience given her son had a smartphone. Even though she tried to limit her AYA’s time online, she also promised to respect his autonomy by letting him set the tone for his information needs:

I tried to Inot let him get online very often, which is very hard for a 16 to 17-year-old who’s got a phone. … We were very honest with him about everything. We told him, “You’re an adult almost. You need to know the answers. We’re never going to lie to you. If you have questions, we’re going to try and find the answer together.” (8)

#### 3.1.2. Clinical

While parents often wanted to be the driver of clinical interactions to advocate for their AYA as their caregiver, they also described the importance of their AYA’s need for autonomy from their parent in their healthcare management. This tension, at times, created a tug and pull dynamic during medical encounters:

There’s some things that I want to push to have addressed [in the appointment] and [my daughter’s] like, “No! I don’t want you to do that.” That does occur and again—it’s walking that fine line of advocating but not [being] overbearing. (20)

Parents described how their AYA’s developmental phase or maturity played a role in this paradox:

He’s a typical teenager or young adult. But it’s fine. He is very responsible and has had to become extremely responsible in this whole process over this past year. But yet, he is still a young adult. So, I still need to ask him for some reminders or make sure he goes in and checks to let us know what’s coming up next. (1)

This tension could sometimes be further complicated when parents wanted to be included in their AYA’s care discussions and have some control, which clinicians did not always support, positioning the parent as a passenger:

I got weird vibes from the doctors, and I felt like I was super mindful of I got to remember [my daughter] is an adult. She asks the questions. … I was so mindful not to over-talk or I would literally sometimes kind of raise my hand and be like, “Is now a good time to ask a question?” But it kind of rubbed me the wrong way during some of our conversations. There were certain doctors that wouldn’t even look at me! They would look directly at [my daughter]. As a family, we had so many questions. … Some of [the doctors] were looking at us like, “This is a package deal. … This is Mom, and she’s a worried mom. I’ll have a conversation with her.” But, when you have a young adult, especially when you’re in a children’s hospital, they’re not used to dealing with young adults as much. I felt like I was ousted as a parent, and I didn’t have the support. (13)

#### 3.1.3. Family

Parents shared similar tensions of autonomy–control with health-related decisions made within their family environment. Parents wanted to make decisions that prioritized their AYA’s physical health or survival but also wanted to respect or promote their AYA’s autonomy as “the captain” of decision making. This meant parents sometimes reluctantly took on the passenger role in decision making:

[My daughter] says, “I want to go see the Barbie movie.” Her doctor said, “Well, if you’re going to go try to go like maybe during the day when it’s not as many people in the theater and make sure you wear the mask and do all that.” I would have said, “The movie’s going to be here for a while. It’ll probably eventually be on Netflix or something. Is it worth waiting? Just at least wait till after those hundred days!” … We suggested that [and she] maybe thought about it for a couple of minutes. [Daughter said] “Nope.” Okay, we’ll go Wednesday for the matinee. Maybe it’s not as crowded. … They’ll all have their masks on and I’ll be careful. They know if any of them don’t feel well that day or the day before, they’re not coming. … Realizing, accepting, and welcoming the fact that she’s the captain. (6)

### 3.2. Coping with Cancer Together as a Family Versus Separately

Parents were torn between wanting to cope together as a family and the realities of coping apart from one another as individuals. This paradox manifested in two ways. Parents wanted to be present and communally cope with cancer together but circumstances forced family members to be absent and cope apart. Parents also shared instances in which individual coping needs conflicted with the collective family’s ability to cope. 

#### 3.2.1. Online

One parent described this tension emerging with online cancer information. They wanted to manage information as a family. However, coping together with the overwhelming and often negative nature of online information as a collective family unit could be counterproductive to an individual family member’s coping needs in that moment:

Occasionally [we are] approaching it with the appropriate frame of mind, both for us and her, of being able to take that information in without it being preachy or judgey, but an open [approach like], “Here, this may be something you want to know about.” It’s hard to find that balance because it depends on how everybody is physically and mentally feeling, so finding the appropriate moment to do so is challenging. (20)

#### 3.2.2. Clinical

As a cancer caregiver, parents wanted to be present in clinical settings to support their child but for some discussions, as a parent, they wished they were absent. These included encounters about issues that parents and AYAs would not typically have discussed, like their AYA’s fertility and sexual behavior. They wanted to be present to provide caregiving support, yet also absent to protect their child’s privacy or reduce discomfort:

I didn’t feel awkward about [talking about sperm banking], but I felt for him because he even said, “That was really embarrassing.” When he’s going in that room and doing that, [it] is super embarrassing. I felt for him because it is! What kid does that? And they wanted me to ask him some private questions and I’m like, “I think he probably has it under control.” (10)

Parents also shared a coping paradox when individual approaches to clinical information collided and took precedence, thereby inhibiting communal coping. Parents and AYAs at times differed in the amount of information desired during clinical encounters. This became more complicated during the COVID-19 pandemic:

I’m definitely an information seeker. … This was also during COVID … so [during] that first phase of him being in the hospital, we weren’t allowed to visit him. It was very hard. That added a layer of complexity of trying to get information to talk to people because it was only when the doctors would come in and he would FaceTime us and so it was about five minutes a day that we had time to actually talk to people there. Otherwise, I was reaching out to the nurses to check on him to find out what was going on to get more in-depth information [because my son] was good in communication, but he didn’t want to know or didn’t care about the details—the level of details that I wanted to know. (4)

#### 3.2.3. Family

In the family setting, parents described multiple ways the tension between coping together and apart emerged, both as an individual–collective tension and absence–presence tension. For instance, at times, parents’ desire for communal coping clashed with individual coping responses. In such instances, they prioritized their AYA’s individual needs over others’:

It was more so me breaking down than [my son] did. His attitude towards this really made this situation. … I wanted to do the whole breakdown thing, the pity party, … the “Why is this happening?” … So, for me, it was more so trying not to break down in front of him and trying not to feel because he had the right attitude. I just needed to get on board. (14)

Additionally, parents shared that because they needed to be present and cope together with their AYA as their parent and primary caregiver, this competed with other roles, like being absent from parenting their other children:

I remember just feeling resentful that I had turned my life upside down and moved, and I did not have time with my [other] daughter, and she was struggling. So, there were times when I was listening for is this a time that I can go home and be a mother to my daughter? Is it safe for me to do that? Which of course I didn’t ask directly because [my diagnosed son] needed me to be there. He did not need to be responsible for my experience. (17)

Finally, parents wanted to cope as a family by being present together, yet, physically could not due to lost shared time: “We were running like separate, almost separate lives. Like if I was at work, [spouse] was at the hospital. If he was at work, I was at the hospital” (2). When a parent and AYA had to relocate for treatment, this tension was further challenging and fraught with emotional and physical distance as they struggled to connect as a family system:

When we’re together, we don’t really have a family life right now. … The last time we did something together, all four of us, was right before [older son] left. We spent three days together, and it was horribly awkward because the boys don’t know how to act around each other, because they haven’t been together, and they’re 15 months apart! And my husband and I are like best friends who happen to be married. We don’t talk about finances. We don’t talk about normal marriage things. He just knows he has his role. I have my role, and let’s not burden each other. … It’s affected everything. I mean, we’re not a four-part family anymore until maintenance [therapy] maybe hits because we’re so distant. There’s so much distance, physical distance in between us. Yes, we make the phone calls every day, but it’s just different. (9)

### 3.3. Weighing Whether to Reveal Versus Conceal Information

Parents wanted to reveal or share disease-related information with their child and, thus, be open, to protect their AYA’s physical health and promote their long-term survival. Concurrently, parents wanted to withhold distressing information, or be closed, to protect their child’s current psychological well-being. Parents reflected on a paradox between prioritizing their AYA’s physical versus psychological health needs.

#### 3.3.1. Online

Parents struggled with wanting to openly share online information they perceived as potentially powerful knowledge in their fight against cancer. Still, information was also perceived as potentially psychologically harmful by inducing fear or sadness. Thus, parents also wanted to conceal information to buffer their child from distress:

It’s kind of a two-way street—trying not to share things that are going to be unnecessary or overly negative and not helpful but, in the same time, when there is something, having an open conversation of, “We found this. You’re welcome to go here to look at it for yourself.” It’s just one of the things that we’re keeping that communication open. It’s her life. It’s her health. We just want to be here to support and help guide. (20)

#### 3.3.2. Clinical

In clinical settings, parents encountered a similar tension of wanting to share information with the clinician to better ensure their AYA received quality care. In contrast, AYAs wanted their parent to withhold information they viewed as private or vulnerable or that disclosure was not necessary or pertinent. This information was related to physical symptoms, mental health issues, logistical challenges, and personal matters:

I think the one time I did irritate [my son] was when he went on his honeymoon, and I mentioned that he was going out of the country. He wasn’t going to bring that up! [I said to him] “Are you kidding? Are you kidding me? That’s kind of an important thing that you’re leaving the country! I’m nervous for you!” (4)

#### 3.3.3. Family

Within the family environment, parents worked to keep communication open with their child to provide emotional support as their primary caregiver. However, parents’ openness competed with AYAs’ avoidance, which parents attributed to their developmental phase in life:

I think [son] and I have been through something together that’s really important. I would not say that we’re closer. … He’s just kind of got his head down right now getting through the mental health aspect of remission, but he is holding a lot of things closed right now and not sharing a lot. He is seeing a social worker, so some of that is cancer, but … [he’s a] 19-year-old too because who wants your mother in every aspect of your life when you’re in college? (17)

Parents also shared how the reveal–conceal tension emerged in their communication with other family members. They wanted to be open with family about their AYA’s cancer experience to obtain support themselves, but their openness was sometimes met with closed responses from family resistant to hearing the disclosures:

My one sister pretty much let me down, but she apologized later. She went dark on me for a while. I know one time we were in the hospital and there was a baby, like a one-year-old. He was by himself. He was crying nonstop across the hall, and I asked the nurses, like, can I just go hold him? Like, he’s alone. He’s here. He’s scared and I couldn’t because of COVID. And I tell that story and my oldest sister then lashes out at me, like, "Quit telling us these horrible stories." It made me feel so alone. That’s when I learned I could tell my mom things, but not them. (10)

### 3.4. Expecting Normative Developmental and Disease Trajectories Versus Disrupted Trajectories

Parents described two related paradoxes that evolved from disrupted expectations. Parents and their AYA struggled with trying to have a typical developmental trajectory, or what would be expected as an adolescent/young adult, versus the reality of being an AYA with cancer—an unexpected life event and atypical experience early in the lifespan. Parents also struggled with disrupted expectations of the cancer trajectory: they encountered a tension of wanting/expecting stability as their AYA transitioned to survivorship while unexpectedly facing ongoing instability. Parents described these tensions characterizing their clinical and family communication experiences.

#### 3.4.1. Clinical

In clinical settings, parents described the conundrum of an AYA not having the typical maturity needed to navigate oncology care while needing their child to be more mature because of their diagnosis:

He’s a young adult. You know, young adults are not supposed to be going through these things! [But] I mean, you could get blown away by the things that could be provided and done to you without proper education and without asking proper questions. It’s not something you should go into blind. (5)

Parents wanted their AYA to be more mature to promote their survival during this atypical experience, yet, recognized their ability was limited given their age: “His using his voice is so important but, at the same time, on the exact same note, a 17-year-old boy can only get to do so much” (9). 

Additionally, parents shared how clinically they expected stability in their AYA’s health status once they ended active treatment or entered survivorship. However, their cancer trajectory expectations were disrupted and their expectation for stability was met with ongoing instability and an uncertain future:

Everyone thinks it’s just a switch. I thought the same thing. Treatment will be over. We get to resume normal life like it never happened. … That’s not the way it is. Secondary cancers can come from all [the treatment]. And [there are] other things that we have to constantly be on the lookout for and all the organ damage. (10)

#### 3.4.2. Family

Parents also wanted their AYA to have typical socioemotional experiences as a developing adolescent or emerging adult, but developmental milestones were disrupted due to the cancer diagnosis (e.g., “He’s missed most of his high school experience.”). While parents wanted their AYA to have typical social experiences like time with peers, that desire sometimes conflicted with prioritizing their AYA’s survival. A parent explained this paradox regarding her newly married son who was now living with them: “You want your newlyweds to go thrive and go on and live your life! … That’s the hardest part to watch. … He just needs to take care of himself right now. He’s not focused on anything else” (4).

Parents also shared that sometimes they needed to encourage their AYA to engage in typical social activities to promote their ability to have some normative experiences in the midst of their atypical reality of living with a blood cancer and loss of other typical developmental milestones:

Four of his friends on spring break went on a guy’s trip. I kind of forced him to go. He didn’t want to go. He just kept talking himself out of it. But I forced him. I was paranoid, too, at first! I can’t let him go six hours away without me! … [He went and] I was on the phone with him when he needed it. … It was such a great thing for him to do! He managed all the symptoms. … [Over the phone] I didn’t talk about cancer once or how he was feeling—nothing. I just wanted to hear what they were doing. I took the focus away from that. In the meantime, … there was a lot of drama with his school. He just wanted to walk at graduation with his friends. He didn’t have enough credits because he was behind. … They wanted him to finish all these assignments the last two weeks before graduation. There was no way he could do it. … He was managing all this mental and emotional pain that he was going through, and then they tried to give him all Fs in his classes. They caused so much other stress and pain in our life. … I finally got them to remove the Fs, and I withdrew him, so it didn’t count against him. But they didn’t let him go walk at graduation. … He had a pretty good summer. … They did have one more guy’s trip in August. … That, again, was awesome. A really great experience for him. … It was nice to see him doing normal things. But when he got home, he did tell me, he said one day I had to sleep the whole day. I was so exhausted. He tried to keep up with his friends, but he can’t. (10)

As parents helped their AYA navigate the ongoing disruption to their developmental trajectory, parents described how their AYA faced ongoing instability even during transitions in which they expected stability to resurface. As their AYA transitioned to survivorship after active treatment, they shared how their expectations were disrupted yet again, thereby prolonging instability:

I think a challenge is that you expect, well, now [son’s] home, and he’s in remission and he’s all better. And it’s a challenge to know that he is not better. He’s not 100%. He still is weak. … I think that’s a challenge—just knowing that he looks good on the outside. He’s going to tell you he’s good. You have to know that his body is still really recovering. … We’re not able to do everything that you used to do. (2)

## 4. Discussion

Parent caregivers of AYAs diagnosed with a blood cancer constantly feel pulled in two, opposing directions as they navigate their cancer caregiving and parenting roles in online, clinical, and family care contexts. These tensions illustrate the difficult dialectical “dance” parents must learn to navigate after their child’s cancer diagnosis as they weigh dual, conflicting perspectives that co-exist within a given caregiving situation. Collectively, these tensions demonstrate that parents’ priorities as a cancer caregiver (i.e., helping their child survive cancer and receive quality care) can compete with their priorities as a parent (i.e., nurturing and parenting their child to have typical human development including shielding them from distress). 

Dialectical tensions experienced by caregivers can be informed by the type of caregiving relationship or relational dynamics (e.g., parent of a child/intergenerational relationship), type of disease (e.g., blood cancer), and developmental context (e.g., AYA) [10,19,38,39,40,41,42]. In particular, parents of diagnosed AYAs struggle with dilemmas of *control and autonomy* in their child’s care, wanting to lead clinical conversations while also wanting to respect and foster their son/daughter’s growing independence as their parent. They also are challenged by tensions of *presence and absence* in their *individual versus communal* coping with cancer that is, at times, determined by caregiving demands, which can separate family relationships emotionally and physically. Parents also constantly balance *openness with closedness*, as they frequently make decisions about sharing or withholding information and the benefits to their child’s cancer care versus the risk to their emotional state. At the same time, parents are in a fight for the *typical versus atypical* or *stability versus instability* as they long for typical age-related experiences for their child with peers and education, while wanting to prioritize their child’s survival in both care and parenting decision making. 

Results demonstrate that parents encounter and navigate the tug and pull of these challenging forces across online, clinical, and family care contexts, indicating the consistent nature of tension in all facets of caring for an AYA with blood cancer. It is noteworthy that these tensions can emerge in each context and, yet, parents’ management of each tension may manifest differently depending on the communication context and parenting or care goals. For instance, while parents want to be open in clinical communication encounters to promote quality care and survivorship (a caregiving goal), they may be less open in sharing online information about cancer to buffer their child from distress (possibly a parenting goal). At home, parents may want more openness than their adolescent or young adult child is willing to give (for both parenting and caregiving motivations), as their AYA engages in more avoidant behavior with their parent relationally at this phase of the lifespan.

Collectively, these findings can be used in developing psychoeducation and supportive care resources in AYA oncology. For instance, psychotherapists in private practice and oncology settings (e.g., psychologists, mental health counselors, licensed clinical social workers) may utilize a dialectical behavioral therapeutic approach that brings awareness to the tensions identified in this study and normalizes the experience of feeling simultaneously pulled in two directions when caring for their AYA. Programs for individuals and families can be developed that incorporate DBT skills for emotional coping and communication (e.g., see [43]) and that normalize parents’ opposing feelings—that it is possible to think, feel, and act in more than just one way—as their child’s parent and caregiver [44]. Supportive groups or individual therapy could also incorporate reflective written activities (like a caregiver workbook [44]) to help parents process their feelings related to each side of the tension, which can be used to talk through constructive ways to adaptively navigate them. Furthermore, online self-paced caregiver tools, like the *Healthy Communication Practice*, can be adapted to incorporate these findings and used by caregivers on their own (or in conjunction with supportive cancer care in AYA programs) to help parents develop communication skills for navigating the tensions in online, clinical, and family care contexts—skills that will also enhance their decision making [29]. These resources are imperative as evidenced in psychotherapy-focused research and psychosocial coping research, both of which have both demonstrated better coping and health outcomes when individuals facing traumatic experiences like cancer better understand the dual forces that define their illness experience [14,15,16]. In understanding these tensions and normalizing them, studies show that caregivers, family members, and patients can adopt more flexibility in how they cope with illness and care-related stressors, which promotes better health outcomes like emotion regulation, reduced distress, and better relational functioning [16]. 

Findings can also be used in medical education for clinicians to educate them on the challenges that parents are grappling with so that they can better understand these dualling dynamics during clinical encounters. A dialectical framework has been argued for in nursing practice to help clinicians make sense of clinically complex experiences when more than one contradictory view exists within a situation [38,39,40]. In knowing the tensions parents are managing, clinicians can better equip parents in their caregiving role and, at the same time, better approach challenging situations that may arise within clinical encounters and medical decision making in triadic clinical communication (between AYA–parent–clinician).

### Limitations

Parents were mostly White mothers; further research with racially and ethnically diverse parent populations is needed to fully address differences related to culture that may inform parent–child dynamics and relational expectations. Furthermore, while mothers often take on primary caregiving responsibilities for a child with cancer, fathers’ voices are imperative to capture, as are dyadic perspectives. A recent study indicated that recruitment materials that include father-targeted materials versus only parent-targeted may enhance recruitment of caregiving fathers [45]. Additionally, given the financial hardship families face and disruption to their lives separate from cancer (e.g., having to relocate, leave jobs, pay for lengthy treatments), families representing diverse socioeconomic backgrounds should also be heard from to provided targeted guidance. Partnerships with targeted recruitment with oncology social workers may enhance the ability to identify families facing such hardships. Future research should also include the diagnosed AYA’s perspectives to triangulate parent–child findings and broaden our understanding of dialectical tensions that occur within this relationship. 

## 5. Conclusions

Parent caregivers of AYAs diagnosed with a blood cancer must navigate tensions or competing needs in online, clinical, and family care contexts. Psychosocial education and interventions that teach parents about and normalize these tensions may help them manage them in ways that promote supportive parent–child connections. Considering caregiver–patient outcomes are interrelated [2,3,4], such educational resources may contribute to the reduction of distress for both the parent and their diagnosed AYA.

## Figures and Tables

**Table 1:** Parent caregiver and AYA patient demographics.

Characteristic	*n* (Percent)	Mean (SD)	Min-Max
Age			
Caregiver		52 (6.76)	38-69
Patient Age at Diagnosis		19.9 (3.99)	15-28
Patient Developmental Group			
Adolescent	10 (50%)		
Emerging Adulthood	10 (50%)		
Caregiver Gender			
Female	19 (95%)		
Male	1 (5%)		
Patient Gender			
Female	5 (25%)		
Male	15 (75%)		
Caregiver Race/Ethnicity			
White, non-Hispanic	16 (80%)		
White, Hispanic/Latino	2 (10%)		
African American, non-Hispanic	1 (5%)		
African American, Hispanic/Latino	1 (5%)		
Caregiver Employment Status			
Employed, Full-Time	10 (50%)		
Employed Part-Time	5 (25%)		
Not Employed/Retired	5 (25%)		
Patient Diagnosis ^1^			
Leukemia	15 (75%)		
Lymphoma	5 (25%)		
Patient Diagnosis Subtype			
Acute Myeloid Leukemia	4 (20%)		
Acute Lymphoblastic Leukemia	9 (45%)		
T-Cell Leukemia	1 (5%)		
Hodgkin’s Lymphoma	4 (20%)		
Non-Hodgkin’s Lymphoma	1 (5%)		
Diffuse Large B-Cell Lymphoma	1 (5%)		

^1^ One AYA was diagnosed with both leukemia and lymphoma.

**Table 2:** Tensions parents encounter while caring for their AYA with blood cancer.

Caregiving Parents of AYAs Need Help Managing These Four Tensions in Which They Feel Simultaneously Pulled in Opposing Directions:	Parents Experience the Tension in Multiple Care Communication Contexts	As Each Narrative Illustrates:
	online	I tried to not let him get online very often, which is very hard for a 16 to 17-year-old who’s got a phone. … We told him, “You’re an adult almost. You need to know the answers.”
being the driver vs. a passenger in their child’s care	clinical	We have to be like, “Yes. I would love to be involved in everything.” However, she is a young adult, and I have to respect that choice.
(a tension of control–autonomy)	family	[You] just accept … the fact that she’s in charge and give her all the support that you can possibly give, even if you don’t agree with it. Now, obviously if that’s detrimental to her well-being, you got to say something or step in! If [doctors] say, “Don’t eat sushi,” and [son] goes, “I want sushi, damn it. And go get it for me.” No, you got to draw that line.
	online	Occasionally approaching it: “Here, this may be something you want to know about.” It’s hard to find that balance because it depends on how everybody is physically and mentally feeling, so finding the appropriate moment to do so is challenging.
coping with cancer together as a family vs. separately/individually	clinical	He’s ready to be done with this whole, with the hospital setting stuff. So, he might rush it more [with clinicians], whereas I might say, “But can you talk to him more about whatever?”
(tensions of presence–absence and individual–collective)	family	There’s times I know she wants us there. I know she doesn’t like when we’ve come home, but she’ll even say, “You guys go home. You got to go take care of this, … cut the grass and you have the dog and you have the cat and you have your meetings.” … But you know, she’s not happy when we’re not there.
	online	It’s kind of a two-way street trying not to share things that are going to be unnecessary or overly negative and not helpful, but in the same time when there is something having an open conversation of, “We found this. You’re welcome to go here to look at it for yourself.”
weighing whether to reveal vs. conceal information	clinical	He ripped his port bandage [golfing]. … Instead of saying, … “Maybe it was from sweat.” No, [we should tell the doctor that] he was golfing. … He’s like, “Mom! It looks like I’m being irresponsible.” Well, they just need to know. I don’t know. So, being too honest [with the clinicians] has created tension.
(a tension of openness–closedness)	family	For example, my daughter is very much of a - I just need to know what you know [personality]. [But] I think maybe it’s hard for her to hear some of the rougher details of, okay [her brother] had diarrhea today or he threw up or he’s [having] fevers.
expecting/wanting normative developmental and disease trajectories vs. facing disrupted trajectories	clinical	Young adults are not supposed to be going through these things, … [and] it’s not something you should go into blind. And for somebody like him, his level of education and his mentality, he would not have been able to … figure it all out on his own.
(tensions of typical–atypical and stability–instability)	family	We hate it for them because they’re newlyweds, right? And you want your newlyweds to go thrive and go on and live your life. … [But] he just needs to take care of himself right now he’s not focused on anything else but that so that’s hard to watch.

## Data Availability

The data presented in this study are available on reasonable request from the corresponding author with IRB approval. The data are not publicly available due to participant privacy.
